# Shifts in evolutionary balance of phenotypes under environmental changes

**DOI:** 10.1098/rsos.220744

**Published:** 2022-11-02

**Authors:** Maria Kleshnina, Jody C. McKerral, Cecilia González-Tokman, Jerzy A. Filar, James G. Mitchell

**Affiliations:** ^1^ Institute for Advanced Study in Toulouse, Toulouse, France; ^2^ College of Science and Engineering, Flinders University, Adelaide, Australia; ^3^ School of Mathematics and Physics, University of Queensland, Brisbane, Australia

**Keywords:** eco-evolutionary dynamics, execution errors, evolution of cooperation

## Abstract

Environments shape communities by driving individual interactions and the evolutionary outcome of competition. In static, homogeneous environments a robust, evolutionary stable, outcome is sometimes reachable. However, inherently stochastic, this evolutionary process need not stabilize, resulting in a dynamic ecological state, often observed in microbial communities. We use evolutionary games to study the evolution of phenotypic competition in dynamic environments. Under the assumption that phenotypic expression depends on the environmental shifts, existing periodic relationships may break or result in formation of new periodicity in phenotypic interactions. The exact outcome depends on the environmental shift itself, indicating the importance of understanding how environments influence affected systems. Under periodic environmental fluctuations, a stable state preserving dominant phenotypes may exist. However, rapid environmental shifts can lead to critical shifts in the phenotypic evolutionary balance. This might lead to environmentally favoured phenotypes dominating making the system vulnerable. We suggest that understanding of the robustness of the system’s current state is necessary to anticipate when it will shift to a new equilibrium via understanding what level of perturbations the system can take before its equilibrium changes. Our results provide insights in how microbial communities can be steered to states where they are dominated by desired phenotypes.

## 1. Introduction

Despite the primacy of evolution in biology, there remains the critical gap in our understanding of how environment influences evolution [1]. This is especially relevant for environmental changes from the scale of small groups to global climatic events. Moreover, the time scales for genetic adaptation are often slower than environmental changes, meaning that the important response is in the phenotype.

Bacteria are ideal model systems for studying phenotypic response due to their rapid reproduction, comparatively simple biology, and suitability for laboratory study. With most bacterial sensory systems limited to molecular uptake, it is impossible for them to anticipate environmental changes. As the most abundant life on earth, bacteria regulate the biosphere while being intimately connected to it. A single bacterial species, unlike animals, may rapidly change its role from abundant to rare, and from dominant-aggressive to rare-passive or some mix of the two depending on local habitat [2,3]. This may lead to trait variation in originally genetically identical organisms [4]. Microbial research often focuses on cooperation or competition among species, extending experimental findings to evolutionary models [5–11]. The intra-specific interactions are often overlooked. However, it is the within-species phenotypic variation that shapes genetic drift, which subsequently determines the genome evolution behind interspecific interactions.

For identical genotypes in static environments, reaction to stimuli can be distinct [12]. Stochasticity in gene expression occurs in various settings and at different levels, playing a role in bacterial interactions, such as motility [13], genetic competence [14], persistence [15,16], sporulation [17], metabolism [18] and stress response [18–21]. In static environments, phenotypic robustness is more likely to evolve than plasticity, indicating that a stable phenotypic equilibrium will be reached [22,23]. However, under environmental change, gene expression noise may become beneficial for population fitness, indicating that stochasticity probably plays a key role in microbial communities [24].

We aim to explore what might happen to the phenotypic structure of microbial communities under environmental shifts by adopting a stochastic-gene-expression approach. Bairey *et al*. demonstrated that even stable communities can be destabilized by random interactions among species [25]. Hence, the importance of high-order interactions has been studied and recognized. However, the interplay of randomness between exhibited behaviour and environmental fluctuations has not yet been addressed. Frequently, the scale of the environmental fluctuations is taken to be local, due to the environmental feedbacks between microorganisms and concentrations of nutrients [9]. However, besides local environments, microbial communities have to survive and adopt to global changes. In our study, we consider the interplay between global environmental changes and phenotypic bet-hedging in microbes.

This may extend to changing environmental conditions, where variability in fitness between the different environmental states might encourage diversification of gene expression. The lack of phenotypic robustness would then provide bacteria a better chance of managing environmental shifts, as the presence of a phenotype suited to the new conditions would confer higher chances of survival. Our definition of the lack of phenotypic robustness is called incompetency and differs from a common idea of behavioural mistakes. In particular, there is no correction mechanism, and organisms will continually execute the wrong phenotype. In this context, understanding which phenotype is most likely to survive is a key challenge in unravelling the complexities of the evolutionary processes within these systems.

To investigate the properties of phenotypic interactions in evolutionary processes, we construct an evolutionary game accounting for environmental shifts. Evolutionary game theory has been widely applied to biological systems since its emergence in 1973 [26]. Analysis of microbial interactions and evolution within this setting is more recent.

Game theory was applied to study various microbial interactions, such as the evolution of metabolic pathways, public goods production, cross-feeding, cyclic competition, signalling and host-parasite evolution [10,11,27]. When addressing the evolution of populations, the literature focuses on two classic approaches with finite or infinite population size [28], with more recent developments of finite but large populations in [29]. As the goal of this study is to understand the effect of global environmental shifts, we adopt a classic approach of infinitely large well-mixed populations. In such games, the population dynamics are continuous and, hence, smooth environmental transitions can be studied. We also adopt a classic pairwise interaction approach, due to its tractability. For studies on more general *n*-player interactions see [30,31]. While this is a convenient mathematical method sometimes used to provide insights in microbial systems with stochastic environmental fluctuations [32,33], one needs to keep in mind its limitations as taking into account more types can change the result of the competition [22,25,34]. Despite the limitations, given the focus on the global scale of environmental shifts, prior studies suggest that qualitative game-theoretic models can still provide insights into factors that drive the evolution in large communities [9,10,32].

Perhaps the most common setting studied is that of social dilemmas where altruistic behaviour can be exploited by selfish individuals. Yet, cooperation is ubiquitous in nature, and as a result many models are devoted to understanding the evolution of pro-social behaviour [35]. Social dilemmas can be modelled as a two-player game with actions ‘cooperate’ and ‘defect’. While many different versions of social dilemmas exist, most common are games where in a one-shot game either defectors dominate cooperators or there is stable coexistence. The two behaviours are displayed in Prisoners’ Dilemma [36] and Snowdrift/Hawk-Dove game [37], respectively. Prisoners’ Dilemma is characterized by cooperation being difficult to achieve in infinitely large well-mixed populations and additional mechanisms are usually required such as reciprocity or spatial structure. A Hawk-Dove game on the other hand can sustain some degree of cooperation even in infinitely large well-mixed populations [38]. As a result, depending on the exact biological system of interest, different games can be applied and studied under different mechanisms. For example, the game of Hawk-Dove was used to understand microbial public goods production [39,40], cross-feeding [41,42], host-parasite evolution [43], antibiotic resistance [10,44,45] and bacterial wars [20,46].

We will also consider extensions of the two games of interest. In the game of Hawk-Dove individuals can either cooperate or defect. However, even microbes can induce complex social responses such as attack their opponents only when being attacked [46,47]. Such a strategy can be viewed as a retaliation and represents the well-known tit-for-tat strategy [48]. For instance, by adopting tit-for-tat strategy in quorum sensing bacterial populations can sustain cooperation and invade defectors [49]. Furthermore, Prisoner’s Dilemma setting was also shown to promote cooperation when the third strategy, referred to as ‘loners’, was introduced in the community [50–52]. Individuals adopting the loners strategy do not contribute to the community but rather receive a safe (independent on others) reward, which leads to loners dominating defectors. Given that defectors dominate cooperators and cooperators dominate loners, such game settings result in a cyclic Rock-Paper-Scissors-like dynamics, which was observed in microbial communities [49,53,54]. In our analysis, we demonstrate how considering more than two strategies can lead to significantly different evolutionary outcomes when environmental fluctuations are taken into account.

A significant advantage of a game theoretical approach is that it can be constructed in a cost–benefit framework, which helps to determine important trade-offs when studying the impacts of environmental fluctuations and stochastic interactions. Understanding the effect of climate changes on community structures is of a particular importance. While the idea of changes in game payoffs induced by the environment is not new [29,55,56], our study addresses the issue of global environmental transitions at the level of behavioural responses, or in microbial terms, at the level of phenotypic expression. That is, in our model, environmental conditions do not directly change the payoffs, but rather they change individual behaviour via strategies exhibited by organisms. In this case, the effect of environmental transitions can be seen as reflected in a degree of phenotypic plasticity organisms adopt. Given that genetic adaptation may take significant time to occur, phenotypic plasticity often helps to promote biodiversity and adjust to environmental changes [57,58].

In summary, we use a simple modelling framework to try to shed light on the driving factors behind phenotypic competition. For this, we use an evolutionary games setting to explore how this behavioural diversity, captured as the execution of a randomized phenotype, may be beneficial (or detrimental) under different circumstances. Within this framework, we identify which strategies are successful or unsuccessful within static or changing environments, and whether they give rise to stable or unstable systems. Finally, we provide suggestions for future theoretical and experimental work, and place the implications of our work in a broader context.

## 2. Methods

Understanding the complex interactions within microbial communities poses a significant challenge, even before considering evolutionary processes or environmental fluctuations. The ability to deconstruct interaction dynamics in this context comes with computational and experimental difficulties or even impossibilities. Game-theoretic approaches have the advantage of being able to elucidate the macro-scale behaviour of these systems, while requiring few assumptions regarding the underlying drivers of emergent behaviour. In a game-theoretic framework, it is possible to consider dynamic interactions between microbes where the game paradigm, reflecting environmental conditions, changes over time. Such non-steady state settings may then reveal potential shifts in the evolutionary balance within microbial communities, allowing us to suggest mechanisms for detecting which phenotypes survive and which become extinct.

### 2.1. Modelling environmental shifts in evolution

**Figure 1.**
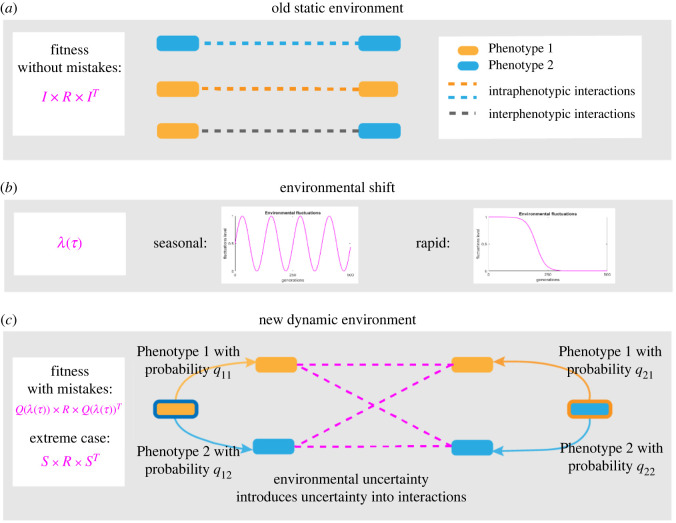
A schematic of the model. We assume that in static environmental conditions intra- and inter-phenotypic interactions are certain and well defined. However, in new dynamic environmental conditions gene stochasticity might lead to uncertainty in interactions. In these new settings, phenotypes may switch. Thus, interactions are no longer predefined, instead being shaped by both environmental and phenotypic expression uncertainty.

We consider behavioural flexibility to be the potential expression of different phenotypes when organisms are exposed to unfamiliar environmental conditions. As this flexibility occurs due to the imperfect realization of the bacteria’s ‘expected’ phenotype, it is referred to as incompetence [59–61] (see [62] for a recent survey of games with incompetent players). Incompetent bacteria may thus change their behavioural traits by switching to a different trait with some probability. The most extreme levels of incompetence are given by a limiting distribution of mistakes, captured by a stochastic matrix, *S*. Matrix *S* expresses the degree of randomization between different phenotypes, that is, gene stochasticity, represented here as incompetent responses (figure 1*a*,*c*). The set of all probabilities *s*_*ij*_ that a microorganism switches its behavioural trait *i* to some other trait *j* can be seen as

2.1 $$S=\left(\begin{array}{cccc} s_{11} & s_{12} & \cdots & s_{1n}\\ \vdots & \vdots & \ddots & \vdots \\ s_{n1} & s_{n2} & \cdots & s_{nn} \end{array}\right).$$

With no incompetence, the matrix *S* is simply the identity matrix. Otherwise, matrix *S* reflects organisms’ responses to a new environment. If new environmental conditions are imposed on the system, the identity matrix *I* (no mistakes, old conditions) may shift to matrix *S* (full incompetence, new conditions). More generally, we may capture these transitions in the matrix *Q*, which describes the change in organism behaviour as they respond to environmental shifts (figure 1*a*,*c*). Matrix *Q* depends on some function *λ*(*τ*), reflecting the environmental dynamics, which we assume are nonlinear. Different types of changes may then be modelled as different functional forms, for example, seasonal fluctuations may be modelled using a periodic function, whereas rapid environmental shifts can be represented by an inverse sigmoid function. This implies the behavioural response to environmental change can be captured in an incompetence matrix

2.2 Q(λ(τ))=(1−λ(τ))S+λ(τ)I,λ(τ)∈[0,1],

where *I* is the identity matrix and *τ* is the environmental time scale. Given fast reproduction rates of microorganisms, we assume that environments change at a slower time scale than microorganisms interactions. This introduces the parameter *α*, which determines the scaling between the reproductive and environmental time scales, that is

2.3 τ=αt.

For the time scale of the environmental change to be slower, we require that *α* ≤ 1. If *α* > 1, then the environment changes faster than microbes interact.

There are two components to our model: environmental fluctuations encoded in *λ*(*τ*) and dynamic behavioural response to the fluctuations captured in matrix *Q*(*λ*(*τ*)). Here, the state of the environment does not depend on the actions taken by individuals. Such decoupling allows for an analysis of higher-order environmental changes rather than specific concentration of nutrients in the media surrounding microorganisms. This, for instance, can be analysed in the framework considered in [63,64]. However, we assume that new environmental conditions impose new demands on organisms, which may be met by using an alternative phenotype. Hence, incompetence can be seen as bet-hedging, such as for instance switching to persistence phenotype that can help the population to survive when exposed to antibiotics [57]. Such settings technically imply that individual microorganisms are capable of adopting mixed strategies, which for instance was observed in baker’s yeast [11]. As indicated in (2.2), over time, there is a distribution of probabilities for the adoption of each available trait, which subsequently affects the reproduction success of different populations. This may be captured within a classic matrix-game scenario, where the fitness of behavioural types is captured in the fitness matrix *R* [26]. Then, under the assumption of a changing environment, we may consider the dynamic fitness matrix defined as

2.4 $$R(\lambda (\tau ))=Q(\lambda (\tau ))RQ{\left(\lambda (\tau )\right)}^{T},$$

where *Q*(*λ*(*τ*))^*T*^ is the transpose of *Q*(*λ*(*τ*)).

Our model can be interpreted in the following way. Firstly, consider pairwise interactions in a given population subjected to new environmental conditions. These organisms have a finite number, *n*, of available strategies, which we refer to as their expressed phenotypes. Hence, interacting individuals compete using their chosen strategies, receiving a payoff according to the fitness matrix *R*. However, we also assume that individuals may be imperfect in their phenotype execution resulting in different phenotype expression according to the probabilities given in the matrix *Q*(*λ*(*τ*)).

As environmental conditions change with *λ*(*τ*), these new conditions may affect phenotypic expression of cells, which we call ‘behavioural errors’ since they do not require genetic mutation. Thus, the errors made by incompetent individuals during their interactions lead to a perturbed payoff *r*_*ij*_(*λ*(*τ*)) from *R*(*λ*(*τ*)). The (*i*, *j*)th entry of the latter is merely the expected payoff that incorporates all possible mistakes made by the two interacting phenotypes *i* and *j*. From the evolutionary perspective, behavioural mistakes perturb population fitness over time as bacteria respond to new conditions. When the environment changes, then bacteria that were incompetent in the old environment might become competent in the new, changed environment. That is, they had deficits in the old environment that are competencies in the new environment. The fitness vector defines reproductive success of each phenotype or behavioural strategy as

f(λ(τ))=R(λ(τ))x,

where **x** is a vector that captures frequency of each phenotype in a population, and the mean fitness of the entire population is defined as follows:

$$\phi (\lambda (\tau ))=x^{T}R(\lambda (\tau ))x.$$

We exploit the well-studied replicator equation [65]

2.5 $${\dot{x}}_{i}=x_{i}(f_{i}(\lambda (\tau ))-\phi (\lambda (\tau ))),\;i=1,\ldots ,n$$

to explore the interplay between evolutionary dynamics and environmental shifts. As stated in equation (2.3), the time scale of replicator dynamics for **x**(*t*) might not coincide with the time scale of environmental change for *λ*(*τ*). That is, environmental shifts may occur faster or slower than the interaction rates expressed by *t*. For microorganisms, however, we assume that environmental shifts happen much slower than their reproduction time scale (that is, *τ* which is slower than *t* or, equivalently, *α* is much larger than 1).

### 2.2. Game settings

We demonstrate the impact of environmental shifts on evolutionary balance using classic examples from evolutionary game theory. We analyse two classic social games: Hawk-Dove (Snowdrift) and Prisoner’s Dilemma. We then extend both these games by adding a third strategy and show how this affects the outcome of the evolutionary dynamics. Throughout the manuscript, to avoid ambiguity, we encode the strategies in the following way: Strategy 1—Phenotype 1, Strategy 2—Phenotype 2 and Strategy 3—Phenotype 3. We also show how environmental conditions impact evolutionary outcomes via the environmental dynamics *λ*(*τ*).

#### 2.2.1. Hawk-Dove

We start with the classic example of a Hawk-Dove game where an aggressive type (Hawk) interacts with a passive type (Dove), which usually results in a stable coexistence of both types. This game is also known as a Snowdrift or Chicken game and was frequently adopted to represent interactions among cooperators and defectors [37]. Assume that there is some resource *b* over which individuals compete. A Hawk meeting another Hawk results in a fight where both individuals have to pay the cost of injury *c*. A Hawk meeting a Dove results in Hawk taking the entire resource and Dove fleeing. Two Doves usually share the resource. Such a game can be described by the reward matrix *R* given by

$$\begin{array}{rl} & \;\;\begin{array}{cc} \;\mathrm{Hawk} & \mathrm{Dove} \end{array}\\ & \begin{array}{c} \mathrm{Hawk}\\ \mathrm{Dove} \end{array}\left(\begin{array}{cc} \frac{b-c}{2} & \;\;b\\ 0 & \;\;\frac{b}{2} \end{array}\;\;\right). \end{array}$$

**Figure 2.**
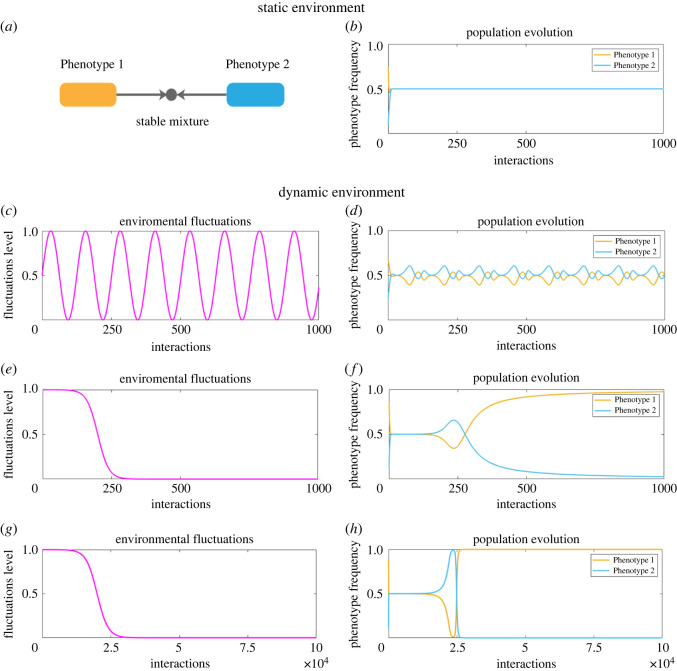
We start with the analysis of the Hawk-Dove game, which for the cost of injury high enough (*b* < *c*) possesses a stable mixed equilibrium of Phenotypes 1 and 2 (*a*,*b*). Under periodic environmental fluctuations, we observe that evolutionary dynamics also exhibits periodic behaviour (*c*,*d*). However, if the environment shifts to a different state and remains there, then Phenotype 1 obtains an advantage and overtakes the population (*e*–*h*). This indicates that the nature of environmental changes might bring different evolutionary outcome. Parameters used to produce the figure: *b* = 1, *c* = 2, *β* = 1/3, *α*_1_ = 0.1, *α*_2_ = 10, *u* = 0.005, *v* = 2000.

In order for the Dove strategy to have any advantage, injuries have to be costly (i.e. *c* > *b*). In our simulations, we set *b* = 1 and *c* = 2. We assume that a Hawk-like strategy characterizes a hypothetical Phenotype 1 and Dove strategy—Phenotype 2. Subsequently, we derive a stable equilibrium between the two strategies (figure 2*a*,*b*).

#### 2.2.2. Hawk-Dove-Retaliator

As an extension of the Hawk-Dove game, we add the third strategy referred to as Retaliator. Retaliators will not instigate conflict but will respond to aggression with aggression, leading to conflicts arising between Hawks and Retaliators. Such a strategy can be seen as a well-known tit-for-tat strategy, where defection only arises as a result of the opponent’s defection. The fitness matrix *R* in this case is as follows:

$$\begin{array}{c} \;\;\;\;\begin{array}{ccc} \mathrm{Hawk} & \mathrm{Dove} & \mathrm{Retaliator} \end{array}\\ \begin{array}{c} \mathrm{Hawk}\\ \mathrm{Dove}\\ \mathrm{Retaliator} \end{array}\left(\begin{array}{ccc} \frac{b-c}{2}\; & \;b\; & \;\frac{b-c}{2}\;\\ \;0\; & \;\frac{b}{2}\; & \;\;\frac{b}{2}\;\\ \frac{b-c}{2}\; & \;\frac{b}{2}\; & \;\;\frac{b}{2}\; \end{array}\;\;\;\;\right). \end{array}$$

Retaliators only escalate the conflict when they meet a Hawk. Otherwise, they share resources with both Doves and other Retaliators. This can be seen as a conditional cooperative strategy. In a classic setting, this is a widely studied game (e.g. [66]), and it has been shown that the game possesses an evolutionarily stable strategy of a mixture of Hawks and Doves. However, it has also been shown that behavioural mistakes might change the evolutionary outcome [60].

#### 2.2.3. Prisoner’s Dilemma

Next, we investigate the effect of environmental change on a Prisoner’s Dilemma, where individuals facing this game have two actions to choose from—cooperate or defect. However, its dynamics tend to a less optimistic outcome than coexistence of both types, because the only Nash equilibrium in this game is to always defect [36,48]. Nevertheless, this game was studied extensively and it was shown that sophisticated behavioural mechanisms can be used to sustain stable cooperation [67]. Usually cooperators pay some amount *c* for the benefit *b* of their opponent. If an individual defects, they do not pay the cost *c*. Such a game can be described by a matrix *R* given by

$$\begin{array}{rl} & \;\;\begin{array}{cc} \;\;\mathrm{cooperate} & \mathrm{defect} \end{array}\\ & \begin{array}{c} \mathrm{cooperate}\\ \mathrm{defect} \end{array}\left(\begin{array}{cc} b-c & \;-c\\ b & \;\;\;0 \end{array}\;\right) \end{array}$$

#### 2.2.4. Rock-Paper-Scissors

**Figure 3.**
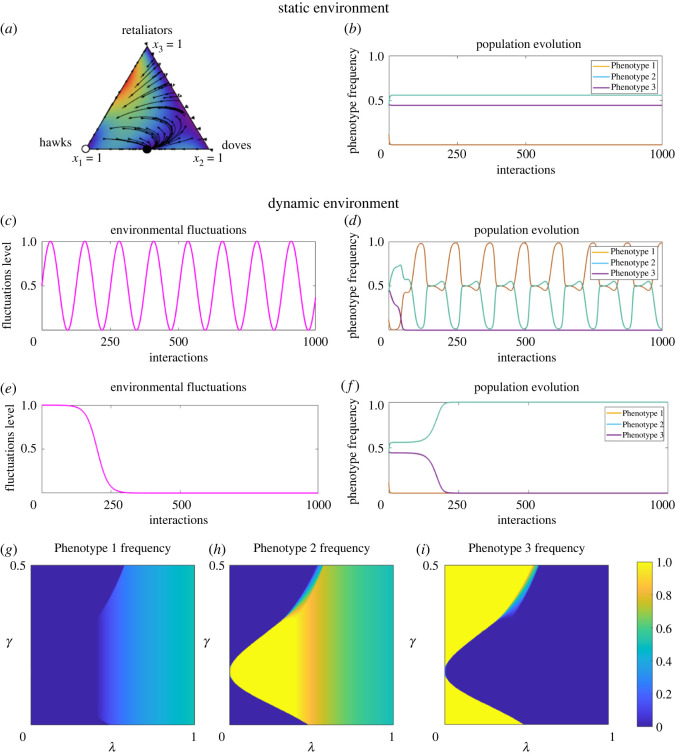
We consider now an extension of the Hawk-Dove game to a three-strategy Hawk-Dove-Retaliator game. In static environmental conditions (*a*,*b*) a stable evolutionary outcome is reachable and, in this case, is a mix of Phenotypes 1 and 2. Seasonal environmental fluctuations (*c*,*d*) lead to a stable periodic solution, where Phenotype 1 is more beneficial. However, rapid non-periodic environmental shifts (*e*,*f*) can destabilize the system, leading to Phenotype 2 dominating the system. Next, we explore the parameters’ space and determine parameters’ values where each of the phenotypes dominate in the Hawk-Dove-Retaliator game. Phenotype 1, which represents Hawks, cannot achieve a higher frequency than 50% for any values of *λ* and *γ* (*g*). However, there exist parameters’ values where either Phenotypes 2 and 3 (Doves and Retaliators) dominate (yellow areas in *h* and *i*). It can be seen that for significant environmental changes (*λ* close to 0), Phenotype 3 has a higher chance to fixate, especially for lower degree of plasticity (*γ* close to 0). Parameters used to produce the figure: *b* = 1; *c* = 2; *γ* = 1/4; *α* = 10; *u* = 0.005; *v* = 2000, initial conditions for (*g*–*i*) are *x*_0_ = (0.2; 0.5; 0.3).

Next, we extend Prisoner’s Dilemma by adding a third strategy called ‘loners’. Loners do not pay the cost, neither do they defect. Unlike others, this type obtains an independent reward. Such a game results in a cyclic competition, since loners always do better than defectors, defectors do better than cooperators and cooperators do better than loners. This results in a game analogous to a classic Rock-Paper-Scissors game, which is frequently used to describe cyclic relationships between microbial phenotypes [53,54,68–70]. It is characterized by inter-dominant strategies (figure 3*a*) with the following fitness matrix *R*

2.6 $$\begin{array}{rl} & \;\;\;\;\;\begin{array}{ccc} \mathrm{Rock} & \mathrm{Paper} & \mathrm{Scissors} \end{array}\\ & \begin{array}{c} \mathrm{Rock}\\ \mathrm{Paper}\\ \mathrm{Scissors} \end{array}\left(\begin{array}{ccc} \;0\; & \;\;-1\; & \;\;1\;\\ \;1\; & \;\;0\; & \;\;-1\;\\ -1\; & \;\;1\; & \;\;0\; \end{array}\;\;\right). \end{array}$$

In this case, Phenotype 1 dominates Phenotype 3, Phenotype 3 dominates Phenotype 2, and Phenotype 2 dominates Phenotype 1. Three different strains locally interact, resulting in an unstable cyclic system where different strains dominate at different points of time. However, to date, this setting has only been observed under stable environmental conditions.

#### 2.2.5. Settings for modelling environmental shifts

From (2.2), we define the limiting incompetence matrices, *S*, to be

2.7 $$\begin{array}{rl} & \begin{array}{cc} \;\;\;\mathrm{Phenotype}\;1 & \mathrm{Phenotype}\;2 \end{array}\\ & \begin{array}{c} \mathrm{Phenotype}\;1\\ \mathrm{Phenotype}\;2 \end{array}\left(\begin{array}{cc} \;\frac{1}{2} & \;\;\;\;\;\frac{1}{2}\\ \;1-\beta & \;\;\beta \end{array}\;\right), \end{array}$$

for two-strategy games and

2.8 $$\begin{array}{rl} & \;\;\;\begin{array}{ccc} \mathrm{Phenotype}\;1 & \mathrm{Phenotype}\;2 & \mathrm{Phenotype}\;3 \end{array}\\ & \begin{array}{c} \mathrm{Phenotype}\;1\\ \mathrm{Phenotype}\;2\\ \mathrm{Phenotype}\;3 \end{array}\left(\begin{array}{ccc} \;\frac{1}{3}\; & \;\;\;\frac{1}{3}\; & \;\;\frac{1}{3}\;\\ \;\frac{1}{3}\; & \;\;\;\frac{1}{3}\; & \;\;\frac{1}{3}\;\\ \;\gamma \; & \;\;\;\gamma \; & \;\;1-2\gamma \; \end{array}\;\right), \end{array}$$

for three-strategy games, where *γ* represents the probability of Phenotype 3, representing loners, exhibiting plastic behaviour as either Phenotype 1 or 2. Such probability distributions provide us an opportunity to compare three abstract phenotypes which display different degrees of plasticity. Hence, we allow both Phenotypes 1 and 2 to be uniformly random in their switching caused by the environment. However, Phenotype 3 represents an additional strategy. Hence, we would like to address the question of the effect of the phenotypic switching of this new additional strategy on the outcome of the evolutionary dynamics. Probabilities of switching are taken to be high enough to reflect critical environmental shifts, that is, those which impose significant stress on the system. However, we still allow for Phenotypes 1 and 2 to express their own strategy with probability 1/3, which would represent completely random switching. We chose such probabilities in order to demonstrate the effect of asymmetric probabilities distributions, where the third phenotype might exhibit asymmetric switching. In any particular case, these probabilities have to be set according to features of the population under consideration. This is of course an arbitrary choice made for demonstration purposes. More general results for any matrix can be found in [60,71].

From (2.2), it is also necessary to define the environmental dynamics *λ*(*τ*), where *τ* represents the time scale of the environmental shift (figure 1*b*). A periodic form of *λ*(*τ*) reflects seasonal or daily regular fluctuations, and may be defined as

2.9 $$\lambda (\tau )=\lambda (\alpha t)=\frac{1}{2}\sin(\alpha t)+\frac{1}{2},\;0<\alpha <1,\;t\in (0,\infty ),$$

where *α* can be interpreted as a frequency of fluctuations in the environmental conditions. For instance, small *α* reflects a longer period after which the perturbation cycle begins again.

However, in the case of rapid environmental shifts, the system moves to a new, fixed state. We demonstrate this principle by extending the model to incorporate the rapid environmental shifts in the form of a reverse sigmoid function

2.10 $$\lambda (\tau )=\lambda (\alpha t)=\frac{e^{-u(\alpha t-v)}}{1+e^{-u(\alpha t-v)}},\;t\geq 0,$$

where *u* > 0 determines the steepness of the above curve and *τ* = *v* ≥ 0 is the inflection point. This function begins with a slow change rate, followed by a steep decrease before levelling off in its approach to the limiting state.

## 3. Results and discussion

We show that an assumption that phenotypic expression depends on the environmental shifts might break existing periodic relationships or lead to formation of new seasonal periodicity in the phenotypic interactions. The outcome depends on the environmental shift itself, indicating the importance of understanding how environmental changes influence affected systems, as well as the exact game settings, such as present strategies, and interaction structure.

### 3.1. Periodic environmental fluctuations

The simplest settings of both the Hawk-Dove and Hawk-Dove-Retaliator games, without errors, are characterized by existence of an evolutionary stable state (ESS) [38,66], where Hawks and Doves coexist (figure 2*a*,*b*), and where the most aggressive species does not have to have the largest population at equilibrium [11]. In addition to the ESS, in the Hawk-Dove-Retaliator game any mixture of Retaliators and Doves is stable in a static environment (figure 3*a*,*b*). However, these equilibria are evolutionary unstable and any perturbation may lead to infeasibility of those equilibria.

We incorporated periodic environmental changes, described in (2.9), into the static models of Hawk-Dove and Hawk-Dove-Retaliator games to explore how periodicity alters evolutionary balance. Such forcing may occur through factors such as temperature changes or nutrient availability through diel or seasonal fluctuations. The adoption of alternate traits under the changed conditions may be a necessity for success or survival [72,73]. We observed that once the changes in conditions are imposed upon the system, the evolutionary outcome may change.

Periodic perturbations in the environment result in a periodic relationship between populations of Phenotypes 1 and 2 in both games, following the same period of the environmental fluctuations. This indicates that stability of the system is possible even in dynamic but regularly changing environments (figures 2*c*,*d* and 3*c*,*d*). Indeed, we prove that there exists a stable periodic solution arbitrarily close to the evolutionary stable state in a static environment (see electronic supplementary material, Periodic forcing). Our results are consistent with classic results predicting that oscillations promote coexistence [11,63,64].

Periodicity in the dominating populations can be explained from the game-theoretic point of view as the organisms’ ability to react to the fluctuations through phenotypic flexibility, which becomes key for survival. The amplitude of the shift in populations varies depending on the rate of change in the environment and phenotype performance levels under the varying conditions. Minor shifts bring only slight change in the phenotypic frequency distributions. However, more extreme changes can destabilize the system and shift the evolutionary outcome to a completely different equilibrium.

### 3.2. Rapid environmental shifts

The second setting of the Hawk-Dove and Hawk-Dove-Retaliator games corresponds to a single, drastic change in the environment. For example, this could be associated with climate change-related events. We apply the function (2.10) to the stable Hawk-Dove and Hawk-Dove-Retaliator systems.

We shall refer to changes in dynamical behaviour caused by the changes in *λ*(*τ*) as switching between regimes, which technically are bifurcations of the dynamical system. A bifurcation value of a parameter in a dynamical system implies that system’s equilibria change their qualitative behaviour or even disappear. For the replicator dynamics with incompetence we define *λ*^*c*^ to be the value where the dynamical system (2.5) changes its qualitative behaviour by shifting stability properties of the evolutionary stable strategy [60]. In this case, it is not only the critical values of the parameter *λ*(*τ*), but also times at which those values are reached that impact the outcome of the game. Hence, we compute the critical time, when these switches occur. The relation between time scales not only determines the rate of response required from organisms, but also their potential to capitalize on their interactions. The system loses its stability at a critical time *t*^*c*^ defined as

3.1 $$t^{c}=\frac{1}{\alpha }\left(v-\frac{1}{u}\ln\left(\frac{\lambda^{c}}{1-\lambda^{c}}\right)\right),$$

where *λ*^*c*^ is the bifurcation value of *λ*. We show that if the environment shifts back to its original conditions, the system is also able to stabilize itself (see electronic supplementary material, Switching between regimes and critical time). Note that a significant effect on the critical time comes from the scaling parameter *α*.

Importantly, critical times may imply that organisms have to rapidly diversify strategies when extreme environmental conditions destabilize the system. Biologically, this would be achieved by bet-hedging, with the presence of some non-dominant phenotypes in the populations, allowing re-emergence of those traits under some conditions [24,74–76].

We assume that environmental changes happen slower than microorganisms reproduce and interact. The replicator dynamics in equation (2.4) capture interactions among microorganisms, hence representing the time scale of evolution. On the other hand, environmental changes happen independently of the interactions and have their own time scale defined as *τ*. Parameter *α* is used as a scaling parameter between these time scales. For example, if *α* is small, the environment is changing very slowly allowing for gradual adaptation and long phase of coexistence. In a potential extension of our model, such long phases can lead to local adaptations and allow for various evolutionary outcomes. In our settings, small *α* might allow for the dominance of a non-aggressive phenotype for some period of time, which can be substantially long, depending on the time scale. However, if the environment keeps on shifting even at the slow scale, the aggressive type will outcompete the non-aggressive type (figure 2*g*,*h*). Large *α* provides a benefit to aggressive phenotypes by allowing them to take advantage of faster environmental shifts without a phase with dominance of the non-aggressive phenotype (figure 2*e*,*f*).

In laboratory settings, one can maintain strictly controlled environments, and could have a very small learning time-scaling constant *α* [77]. Conversely, marine bacteria can be exposed to rapidly changing conditions due to specificity of their environment, such as turbulence [78]. This means that within their bet-hedging, retaining (often) suboptimal strategies may allow those phenotype populations to explode when their strategies become favourable. Such boom-bust dynamics are frequently observed in real-world microbial systems [79].

The trajectory of environmental changes is not the only factor affecting the resulting evolutionary outcome. The construction of the matrix *S*, or the probabilities of phenotype switching, is one of the factors determining the dominating phenotype. Since *λ*(*τ*) encodes the degree of environmental change, it affects the degree of phenotypic switching present in the population. Hence, as *λ*(*τ*) decreases from 1 to 0, the matrix *Q*(*λ*) evolves from the identity matrix (no switching present) to the matrix *S* (the maximal degree of switching possible). As *λ*(*τ*) → 0, the degree of switching between phenotypes, captured in *Q*(*λ*(*τ*)), tends to *S*. In our settings, this implies that Phenotypes 1 and 2 are more likely to coexist, whereas Phenotype 3’s switching follows the probability vector (*γ*, *γ*, 1 − 2*γ*). For such environmental changes, the number of strategies in the game can become essential. For a Hawk-Dove setting, Phenotype 1 outcompetes Phenotype 2, which corresponds to defectors surviving (figure 2*e*,*f*).

In contrast to classic predictions, in the game with three strategies, under the same environmental shift, Phenotype 2 survives, which corresponds to cooperators overtaking defectors (figure 3*e*,*f*) as *λ* decreases from 1 to 0. Nevertheless, there exists a critical threshold for *λ* and *γ*, which provides evolutionary advantage to Phenotype 3 (figure 3*g*–*i*). Note that, according to Phenotype 2 degree of switching, individuals adopting Phenotype 2 will randomly execute any one of the three phenotypes. This preserves all possible phenotypes in the population, allowing for an emergence of a mixed strategy. The separation curves between each pure equilibria stability region are the bifurcation lines that are determined by the parameter values.

The capacity for phenotypic plasticity captured in this model, and our predicted scenarios under which this would be necessary, are reflected in empirical results. Static or slowly changing environmental conditions typically result in lower genomic diversity, whereas exposure to unpredictable and significant shifts promote higher diversity [54].

### 3.3. Shifting evolutionary balance in cyclic relationships

Next, we examine what happens under environmental shifts with a non-transitive relationship already prone to periodicity, as represented by a Rock-Paper-Scissors game. In terms of social dilemmas, such a game can be seen as an extension of the well-known Prisoner’s Dilemma with an addition of a loners’ strategy. It was suggested that loners can naturally evolve as a result of individual cells’ stochastic decision making affected by their environment and signalling of their neighbours [52]. We first examine the effect of environmental shifts in the two-strategy setting.

**Figure 4.**
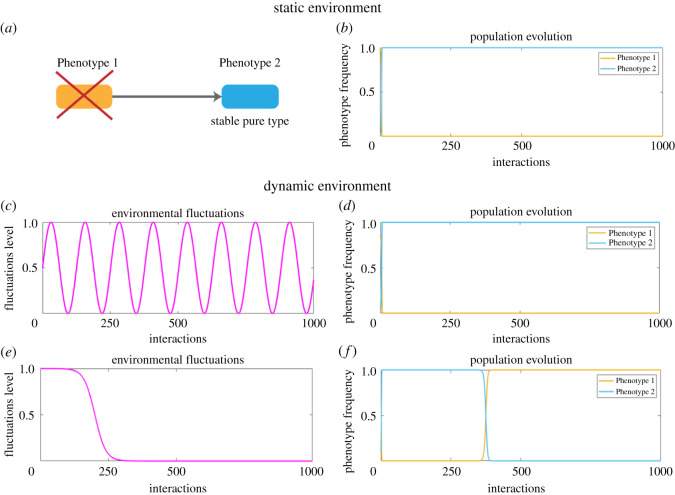
Prisoners’ Dilemma is usually characterized by defectors dominating cooperators (*a*,*b*), even in periodic environments (*c*,*d*). However, environmental shifts and behavioural plasticity may provide an evolutionary advantage to cooperators leading to their dominance (*e*,*f*). Parameters used to produce the figure: *b* = 2, *c* = 1, *β* = 1/3, *α* = 10, *u* = 0.005 and *v* = 2000.

Prisoner’s Dilemma in infinitely large well-mixed populations possesses the only evolutionary stable state where all individuals defect (figure 4*a*,*b*), even in periodic environments (figure 4*c,d*). However, environmental shifts leading to phenotypic switching may disturb such an equilibrium. For a significantly critical shift, cooperators may overtake defectors resulting in a reversed equilibrium while still being able to execute defecting phenotype (figure 4*e*,*f*). However, once the third strategy is introduced in the population, the outcome of the evolutionary dynamics is not as robust and depends on the exact shift, initial conditions and degree of phenotypic switching.

As shown for the Hawk-Dove and Hawk-Dove-Retaliator games, the games which are inherently stable have some resilience against environmental perturbations. However, equilibria in less stable systems of interactions might not withstand significant environmental changes. We demonstrate this on the example of Rock-Paper-Scissors game. This is a popular model in evolutionary game theory, where no strategy globally dominates or is dominated. In a stable static environment, this system exhibits cyclic fluctuations in phenotypic frequencies. In our evolutionary context, the Rock, Paper, Scissors strategies are replaced by Phenotypes 1, 2 and 3, respectively. Such periodicity has been postulated to contribute to variability in complex microbial communities. However, the classic game does not possess an evolutionary stable state, meaning that any perturbation might shift the system out of its equilibrium. This is supported by the fact that non-transitive interactions are rarely observed in natural competitive communities [7]. Furthermore, Müller and Gallas tried to explain why in the experiments on *E. coli* bacteria in the long run only one strain survived [80]. They suggested that cyclic competition is influenced by the exact population size of each strain. We suggest that it might also be due to the inherent stochasticity and phenotypic plasticity [61].

**Figure 5.**
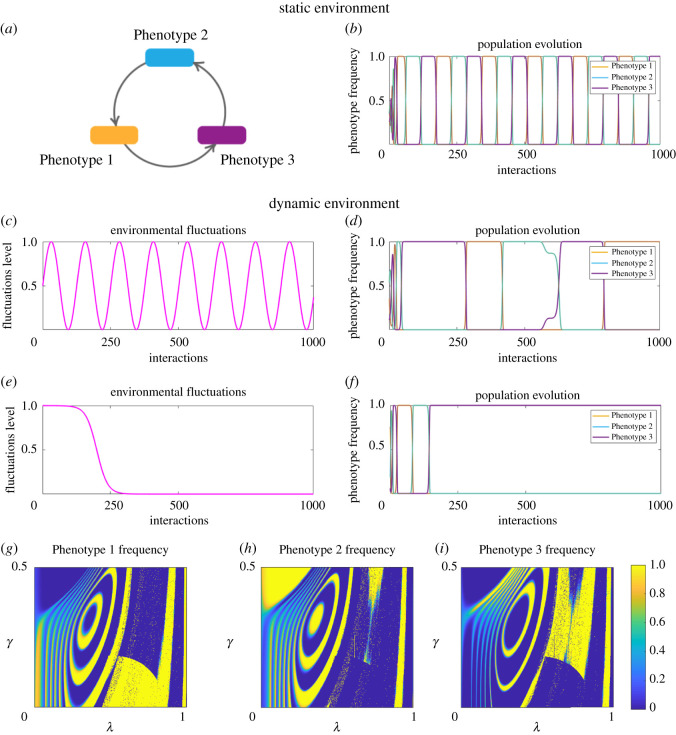
Cyclic relationships, such as Rock-Paper-Scissors, are characterized by periodic fluctuations in the phenotypic frequencies in stable static environments (*a*,*b*) and stable periodic environments (*c*,*d*). Under the assumptions of our model, phenotypic uncertainty together with environmental shifts destabilize the cyclic nature of phenotypic relationships, introducing a winning phenotype (*e*,*f*). However, depending on the interplay between the timing of a environmental shift and the state of the system, the winning phenotype can be any one of the available phenotypes. Same as for the Hawk-Dove-Retaliators game, we perform a similar analysis of the parameters’ space for a Rock-Paper-Scissors game under incompetence. We observe that there exists a periodic character to the resulting equilibria (yellow and blue circles on (*g*), (*h*) and (*i*)). However, we find parameters’ settings, for which one of the phenotypes obtains an advantage (larger yellow areas in each panel). Parameters used to produce the figure: *γ* = 1/4, *α* = 10, *u* = 0.005, *v* = 2000, initial conditions for (*g*–*i*) are *x*_0_ = (0.2, 0.5, 0.3).

In our model, unstable cyclic relationships must adjust to the environmental shifts induced by (2.10). As a result, cyclic dynamics might no longer be in favour as one or another strategy may perform better under the new conditions. Naturally, one would need to determine the state of the cycle where the system was—that is, which of the phenotypes was dominating—when the environment shifted. However, from the evolutionary perspective, this is not the only question requiring an answer. Randomized behaviour resulting in the realization of different phenotypes can shift evolutionary balance of the system to a new equilibrium, where only one flexible phenotype will dominate (figure 5*e*,*f*). However, unlike the cases with stable systems from the previous section, in this unstable case, it is no longer possible to predict which phenotype will assume the dominating position.

As can be seen in figure 5*f*, the system has a limit of perturbations it can take: in the beginning of the environmental shift, the system evolves in a similar cyclic manner as in the old environment. However, the capacity to buffer the shift is not unlimited. Once the system hits a critical threshold, the cyclic relationship stretches its periodicity, and finally stabilizes in the new state, where it will remain. In the case of such an unstable system, in order to understand which of the phenotypes will dominate, one has to examine the interplay between the critical level of environmental changes and the current state of the system. Mathematically, we can predict that the phenotype that is the best response to itself will gain stability, that is, if **s**_*j*_ *R***s**_*j*_ > **s**_*i*_ *R***s**_*j*_ for *i* ≠ *j*, then Phenotype *j* is an equilibrium [61], where **s**_*j*_ is the corresponding row of matrix *S*.

We provide a similar analysis of the evolutionary outcomes as for the Hawk-Dove-Retaliator game in figure 5*g*–*i*. Here, for most environmental changes and degree of phenotypic switching, the relationship between phenotypes remains cyclic (see figure 5*g*–*i*, cycles in the middle of each plot). However, there exist regions of stability for each phenotypic variation (represented by bright yellow areas). Depending on the exact environmental change and degree of randomness of phenotypic switching, each phenotype has a chance to dominate the population. Given that the critical level of changes is hard to estimate in real microbial systems, determining the switching point as a function of time also becomes challenging. Nevertheless, we propose that detection of such switching points might allow prediction of how the system equilibrium will shift under certain environmental changes. For this to be done, experimental studies will be required.

### 3.4. Comparing the incompetence model to existing models

Studying the effects of environmental changes on the evolutionary dynamics has led to the emergence of the eco-evolutionary branch of evolutionary game theory. Among the first papers addressing eco-evolutionary dynamics was [63], following generalization in [64] and extensions to different scenarios of a public goods game in [81,82]. While these studies focus mostly on the evolution of cooperation, many recent studies tried to address a more general set-up for the environmental feedbacks and payoffs evolution [29,55,56]. Our model differs in that it allows for studying the effect of behavioural flexibility, or phenotypic variation, that is driven by an environmental change. Thus, the evolution of the environmental state is independent of the state of the population, which allows us to study the effect of global environmental shifts that are independent of the interactions between organisms. Such shifts can be viewed as anthropological or ecological effects (e.g. climate change) or seasonal and daily fluctuations. This formulation allows for the analysis of the interactions among phenotypes and the effect of the environmental conditions. Under this approach we are able to compare effects of environmental shifts on the phenotypic diversity. We show that degree of plasticity may affect the ability of phenotypes to compete in different environmental shifts.

In a static environment, stable microbial communities can be observed [22], which is a starting point for our analysis for *λ* = 1. This is also true for small environmental fluctuations (*λ*(*τ*) close to 1). However, when environmental fluctuations are periodic, our results suggest that a stable periodic orbit can be observed as was previously shown in [63,64]. In both of these models, it was assumed that individuals affect their environmental conditions, which in turn gives rise to environmental feedbacks. Furthermore, their analysis reveals that cooperation cannot be sustained if the game is a Prisoner’s Dilemma. Our results suggest that in periodic environments a Prisoners’ Dilemma also cannot sustain cooperation. However, rapid environmental shifts can lead to the dominance of cooperators over defectors.

Kussel and Leibler compared stochastic phenotypic switching to sensing followed by a response to environmental fluctuations [57]. They suggested that phenotypic switching can be favoured over sensing when environmental changes are infrequent. They considered two possible types of phenotype switching: as a stochastic random process or as a direct reaction to the environmental stimuli. Kussel and Leibler suggest that organisms that adjust their switching according to the environmental stimuli have a higher chance to survive. Yet, both types of switching are observed in microbes. In our model settings, we consider a mixture of these two: the phenotypic switching is induced by the environmental change. Organisms themselves fail to read environmental stimuli, which results in the stochastic response to changing environmental conditions.

## 4. Conclusion

Evolutionary and ecological theories are powerful approaches for understanding and predicting evolutionary balance of complex systems. In these systems, stochastic gene expression is often treated as experimental noise, leading to its effects being overlooked. However, in dynamic non-stationary environments stochastic gene expression can be advantageous. Here, we used two complementary models capturing periodic environmental fluctuations and rapid environmental shifts and explore gene expression stochasticity in the framework of incompetence, or behavioural errors. With these models we investigated the interaction of phenotypic stochasticity with environmental dynamics.

We analysed the two most well-studied forms of social dilemmas and their extension to three-strategy versions. In static environments, Snowdrift- or Hawk-Dove-like interactions can sustain coexistence of cooperators and defectors even in a three-strategy game, when tit-for-tat players join the competition. However, environmental changes might shift this balance. We reproduced the classical dynamics of the Hawk-Dove-Retaliator game. In a stable environment, the Retaliator loses. With a rapid environmental shift, however, the Retaliator wins. Interpreting this from the microbial ecological perspective indicates that in the case of environmental uncertainty the dominant phenotype may change to one that did not initially appear evolutionarily competitive.

Besides Snowdrift-like interactions, the most common social dilemma, the Prisoner’s Dilemma, makes cooperation challenging to sustain. However, rapid environmental shifts can provide an evolutionary advantage to a cooperating strategy. We also extended this game to a three-strategy game by adding loners to the competition. Such a three-strategy game can be captured in a classic Rock-Paper-Scissors setting of well-mixed infinitely large populations. The inherently unstable dynamics of the Rock-Paper-Scissors game are particularly appropriate for bacteria because of individuals’ metabolic flexibility and their ability to swap genes and so change their phenotype. Again, we reproduced the tripartite, alternating domination result. However, we showed that a single environmental shift can lead to one strategy or phenotype achieving continual domination globally. Our findings might apply only at a global level of populations and their environmental conditions, as at the local level organisms can interact differently with their environment (e.g. [83]).

Overall, microbes are the most suited species to demonstrate the Rock-Paper-Scissors dynamics due to their inter- and intra-generational metabolic flexibility. Also, this model aptly reflects and informs microbial dynamics, where the dominant phenotype can change rapidly, leading to complex evolutionary dynamics and strategies. To understand future shifts in changing environments, we need an understanding of the interaction between gene stochasticity, represented here as incompetent responses, and environmental dynamics beyond the response to a rapid environmental shift presented in this manuscript.

As an additional implication of our results, one can discuss the possibility to drive microbial interactions in a desired direction. Given the short time scales of microbial interactions, the effect of environmental feedback loops raises the possibility of steering the evolution of microbial communities [31,84,85]. In such cases, environmental input encoded in the functional form of *λ*(*τ*) is a control that influences phenotypic expressions of microbes. Being able to predict the response of organisms to global changes in the environment may therefore permit the structure of the community to be controlled in such a way that the desired phenotype becomes dominant.

## Data Availability

The data are provided in electronic supplementary material [86]
